# Social determinants of health: critical consciousness as the core to collective impact

**DOI:** 10.3389/frma.2023.1141051

**Published:** 2023-09-26

**Authors:** Joy Doll, Julie Malloy, Roger Gonzales

**Affiliations:** ^1^Health Informatics, Creighton University, Omaha, NE, United States; ^2^American Occupational Therapy Association, Bethesda, MD, United States; ^3^CyncHealth, Omaha, NE, United States

**Keywords:** social determinants of health, SDOH ecosystem, critical consciousness, Social Drivers, health equity

## Abstract

Social determinants of health have become widely recognized as important to overall health. Many areas of social determinants of health are growing from policy to reimbursement to the connecting of health and social care. The efforts around social determinants of health require reflection and awareness of structural issues. The work of Paulo Freire in critical consciousness provides guidance for how to engage in social determinants of health efforts. This manuscript offers a summary of the social determinants of health under the guidance of critical consciousness to build skills and interactions to promote social care to build toward health equity.

## Introduction

In recent decades there has been a significant focus on the social determinants/drivers of health (SDOH) (Regidor, 2006; Hasbrouck, 2021; Magnan, 2021). In the United States, multiple policy drivers have influenced investments by healthcare organizations to begin to discuss and address SDOH. Despite increased awareness for many, SDOH have always permeated their lives. Structural challenges are not new and where one lives, works, and plays directly impacts health status and utilization of health care services (Plough, 2022). SDOH are “the conditions in which people are born, grow, live, work, and age, including the health system (WHO, 2008)”. It is important to remember that the terminology related to this topic continues to evolve. The term Social Drivers of Health has been proposed in some circles to focus on the fact that these can be changed, and do not necessarily have to be permanent. SDOH will be utilized throughout this document and can be inferred to be either Social Determinants of Health or Social Drivers of Health (Bettencourt-Silva et al., 2020; Doll et al., 2023).

Although there is much discussion on SDOH, there is an unclear path of skills and needed professional development for those working in the SDOH ecosystem. The authors utilize the word “ecosystem” to characterize the work of SDOH that includes many complexities including, but not limited to, community, technology, interoperability, data standards, etc. SDOH efforts require a strong understanding of systems and how the structural aspects of a system impact individual and collective behavior. The line between autonomous choice and the impact of one's context can be easily blurred. For healthcare professionals and others, addressing the individual may not be the solution if the context is directly influencing healthcare outcomes. Individuals working in this space can easily become overwhelmed with the need to change “the system” when impacting the person seems more feasible. However, those in SDOH efforts can look to other approaches and guidance for best practices. Here the authors propose infusing best practices from critical consciousness as a guiding light for those involved in SDOH. It is important to recognize that critical consciousness is not the only opportunity to provide guidance to grow in SDOH work. For example, the work of community-based participatory research provides a framework for community work (Israel et al., 2018). Yet, the authors have found these elements considered foreign in healthcare spaces where terms like reflection more readily resonate with clinicians which is why we propose critical consciousness to support professional development for SDOH work.

## Background on SDOH

SDOH can create inequities and are shaped by the distribution of money, power, and resources at global, national, and local levels. Factors upstream, midstream, and downstream from SDOH are influenced by a multitude of elements such as policy and funding. SDOH also have a major impact on health inequities—“the unfair and avoidable differences in health status seen within and between countries” (WHO, 2022). According to the most recent estimates, the SDOH affect as much as 50 percent of health outcomes in a population (Whitman et al., 2022). All human beings have social needs that are important to survival. However, some humans face structural and social barriers to basic needs such as quality education, health care, safe neighborhoods, affordable and quality housing, social and community networks and economic stability. When these factors are not accessible or if they are of reduced quality, health inequities occur. Health equity promotes that all humans should have the opportunity to be as healthy as possible. On the other hand, health inequities come from poverty, discrimination, and lack of access to basic social needs. When we begin to address health inequities, we can make progress to reduce health disparities and improve the health of marginalized or excluded groups. The health care systems shift from volume to value-based payment has also led to an increased recognition of the impact of SDOH on health care (Adler et al., 2016; Porter and Lee, 2016; Krause et al., 2021).

An SDOH ecosystem is emerging in many regions where existing and evolving resources are connecting in communities to ensure social needs are met. There are two aspects to the SDOH ecosystems that are evolving nationally: partnerships and the activities of those partnerships. Each ecosystem will be unique based on the partners in the community and community need. However, much of the evolvement is based on the efforts to connect health care and social care together, leading to evolving partnerships and collaborations that include community-based organizations, health care organizations, United Way 211s, payers, and social care technology vendors. In some states these ecosystems are emerging as community information exchanges that provide infrastructure to connect health care and social care along with generating data to tell a more informed story around SDOHs. To address SDOH, partnerships that involve stakeholders, champions, and those with lived experience are being created. These partnerships vary based on the local community needs and infrastructure. Important considerations include community readiness, financing, technical tools, data standards, legal resources and governance, policy, and evaluation (Office of the National Coordinator for Health Information Technology, 2022). In this article, the focus will be upon strategies for building collaboration in the SDOH ecosystem to achieve collective action.

A common activity being addressed by SDOH ecosystems is screening for social needs. The screening tools and the individuals who receive the screening vary widely, and depend on the procedures of each organization. Currently health systems decide which tools help them meet the needs of their specific communities, however, this does lead to a lack of consistency in data collection. Screening tools can have a focus on specific public health issues, can vary in the number of questions, can vary in the format of the questions, and can vary in the time it takes to complete. In addition, some healthcare organizations have staff complete screenings, while some have implemented self-screening (Moen et al., 2020). Most of these healthcare organizations have started collecting data surrounding SDOH, but do not have consistent strategies for how the data is saved in the client record, leading to additional inconsistencies and limited use of data (Vale and Perkins, 2022). For those interested in screening tools, the following website offers systematic reviews: https://sdh-tools-review.kpwashingtonresearch.org/ (Kaiser Permanente, 2020).

Related to this, the Joint Commission and Center for Medicare and Medicaid Services (CMS) are implementing a requirement for social needs screening during hospital stays beginning in 2023 (CMS, 2021, 2022a,b; Joint Commission, 2022). This will require discussions about who collects data and how the SDOH challenges that are identified can be addressed. Addressing social care needs can be time-consuming and there may be limited staff allocated to these needs. In addition, some clients may prefer that their SDOH needs not be documented (Galvan et al., 2021; Albert et al., 2022) which results in unmet needs.

While screening is important, it is just the beginning. Resources have to be structured to meet identified needs, and currently these are often addressed by community-based organizations (CBOs). CBOs, are critical members of the SDOH ecosystem, and can include social service agencies, non-profit organizations, and formal and informal community groups [Centers for Disease Control and Prevention (CDC, 2020)]. These organizations help with a variety of social needs like food insecurity, utility assistance and housing. Due to the desire of health care organizations to screen, data platforms that connect CBOs and health systems are proliferating to try to address the gap in communication between these two systems. With these platforms, often called social care referral platforms or community resource referral platforms, comes the call to identify data standards and integration into EHRs to reduce clinician burden and ensure data on social needs is included in the health record (Cartier and Fichtenberg, 2019). Additional areas of growth include resource coordination and navigation as part of the SDOH ecosystem. Becoming familiar with this infrastructure in one's community, including knowledge of the 2-1-1 United Way help line, for example, is critical to build the partnerships and collaboration needed to address SDOH. Data can drive capacity building to expand the services of CBOs by identifying gaps in services and seeking additional resources to address the gaps. In addition, faith-based organizations (FBOs), can also serve similar needs in many communities. These SDOH ecosystems can use data to drive decision making and resource allocation.

Many challenges and opportunities are arising with this new ecosystem. The CMS Framework for Health Equity 2022–2032 (CMS, 2022a) identifies the need “to improve our collection and use of comprehensive, interoperable, standardized individual-level demographic and SDOH data, including race, ethnicity, language, gender identity, sex, sexual orientation, disability status, and SDOH”. To build such an ecosystem for SDOH, a variety of stakeholders are needed to ensure that social care can become a “no wrong door” approach where screening can occur, and needs can be addressed no matter how people enter the system. This calls for stakeholders to be innovative to truly begin to impact SDOH and have discussions across policymakers, payers, public health, providers, and community partners. To help with these discussions the CDC has created a portal with many resources to address SDOH throughout the health care system (CDC, 2020).

In addition, health information technology has a growing role with social care referral platforms. Payment for social care from Managed Care Organizations is growing in some states. Data standards for SDOH are also expanding with the United States Core Data for Interoperability (USCDI) version 3 including more identifiers to support equity through race, ethnicity, and gender identification (USCDI, 2022). The Gravity Project has supported the expansion of *z* codes, which are ICD-10 codes focused on social needs (CMS, 2022b). See Table 1 for resources on data standards in SDOH. The reality is that SDOH are now widely recognized in health systems as important to address. How exactly all of these aspects of SDOH will exist and grow in health care still remains to be seen. Across the nation, the growth and development of SDOH ecosystems are emerging to drive change.

**Table 1:** Resource box.

**Title**	**Link**
United States Core Data for Interoperability (USCDI)	https://www.healthit.gov/isa/united-states-core-data-interoperability-uscdi#uscdi-v3
Office of the National Coordinator for Health Information Technology SDOH Resources	https://www.healthit.gov/topic/health-it-health-care-settings/social-determinants-health
The Gravity Project	https://thegravityproject.net/
ICD-10 Codes	https://icd10cmtool.cdc.gov/?fy=FY2022
CMS Z Code Guide	https://www.cms.gov/files/document/zcodes-infographic.pdf

Data sharing around SDOH is another ongoing discussion in many communities and SDOH ecosystems. Some areas and states have developed what are called community information exchanges that function similar to a health information exchange (HIE) yet are focused on social care data (Vest et al., 2015; Colorado Health Institute, 2021; Grounds and Johnson, 2021; Sorenson and Bloom, 2022). The intent of this infrastructure is to prevent silos of social care data, as has been seen with EHRs. The sharing of data requires considerations around consent, sensitive data, and data governance. Education is critical as many CBOs are new to these efforts and may see an opportunity to use technology as a tool without realizing the implications of data sharing. Data ownership and where data are shared are important discussions to consider in any SDOH ecosystem to ensure health disparities and inequities are not being perpetuated. It is also important to not be naïve that data can be sold and both patients and organizations have a right to know how data will be used.

Despite these challenges, SDOH ecosystems are evolving and growing rapidly. Many communities are coming together to tackle the complexities of social issues, which is long overdue. As this is evolving, a comprehensive approach involving multiple stakeholders is critical. Leading in an organization requires skill building and professional development; yet, it requires even more depth and growth when leading *cross-sector* collaboration. Leaders, partners, and teams building SDOH ecosystems can benefit from understanding the best practices of collaboration learned in the field of interprofessionalism and interprofessional healthcare (Cheng et al., 2020). The next section of this paper will use the best practices of critical consciousness to provide guidance to those looking to lead or collaborate as part of a SDOH ecosystem.

SDOH work is complex, nuanced and evolving. Those working in the field need skill development in order to address the complexities of SDOH work. SDOH efforts require multiple partners and stakeholders to come together to define their priorities and strategies to truly begin to address SDOH. Collaboration and leading partnerships are a core critical skillset for anyone involved in a SDOH ecosystem. Working within a SDOH ecosystem creates and enables inherent conflict. Partners hold different and sometimes opposing priorities. Trust building, clear guidelines and open communication are all critical to ensure success. However, challenges and conflict will occur. These should not be ignored but embraced as an opportunity for all to grow in these cross-sector partnerships and initiatives. Navigating these challenges calls upon the willingness to cultivate a culture of collaboration.

## Critical consciousness as a guiding light

Critical consciousness, a concept introduced by Freire (1970, 2020), is intended to increase awareness of inequities. In fact, without awareness of inequity, the risk is that they carry on in perpetuity. Lack of awareness or experience can contribute to health inequity without intention, making it challenging for health care practitioners or systems to address these structures (Windsor et al., 2022). Based on the concept of critical consciousness, simply increasing awareness of SDOH can be an informative first step to empower addressing inequity. Critical consciousness can be a driver for collaboration, bringing together diverse perspectives to simply recognize that SDOH have an impact on the health status of individuals and communities. It can offer a framework for collaboration that is driven by the dimensions of critical consciousness: reflection, action, and motivation. In fact, despite its academic nomenclature, it is the intentional state of “being critically conscious” that means “the ability to apply knowledge and critical thinking skills to examine current situations, develop a deeper understanding of reality, and generate and implement solutions to problems” (Obiagu and Ajaps, 2022). Grounding collaboration in these competencies driven by critical consciousness can bring important guidance to SDOH efforts to build and sustain a robust ecosystem. The concepts of critical consciousness (i.e., reflection, action, and motivation) provide a foundation for those involved in a SDOH ecosystem.

## Reflection

Engaging teams and partners in reflection is a critical first step to effective collaboration (Sims et al., 2015). It starts with identifying the intent of the collaboration, how partners will collaborate and the overall goals and outcomes of the collaboration. Each partner will have its own assets and challenges along with motivations to be in partnership. Identifying the drivers for collaboration is a key factor to success. Partners may do this in a variety of ways from informal documents like a memorandum of understanding to more formal contracting or a scope of work, often the case if funding is being exchanged. Reflection should occur often and should include a check-in to see if partners are maintaining focus and intention. Some individuals or organizations are comfortable with reflection, honing it as a skill, while others are not. Reflection can be simple—what's going well, what's not going well, and action steps. In addition, reflection can be checking in and giving space for individuals to share their lived experiences in the partnership. Partners may rotate those facilitating reflection or may appoint a member who feels comfortable in this arena. Reflection and sharing takes skill building and time. In Freire's work, he used Socratic questioning as a foundation to drive reflection. He also used the concept of talking circles, common in Indigenous cultures, as a way to elicit reflection and deepened conversation (Diemer et al., 2016). Furthermore, teams and partners that engage in reflection bring depth in interaction promoting the opportunity to delve into issues to drive toward strategies and solutions (Clark, 2009). Recognizing and reflecting can help address these issues in productive conflict to ensure increased engagement and evolvement. In a SDOH ecosystem, asking questions to oneself and others is critical to make sure action can be taken to achieve the goals of the ecosystem.

An important aspect of reflection in critical consciousness is the recognition of the impact of power (Sakamoto and Pitner, 2005). Healthcare organizations and clinicians inherently hold power. Tensions exist between healthcare organizations and community-based organizations around resources and investments. In fact, some healthcare organizations may unintentionally force the competition of resources among the community-based organizations. Freire indicates that due to oppression, vulnerable groups and individuals have adopted silence as a mechanism, particularly those in poverty. As Freire stated “Leaders who do not act dialogically, but insist on imposing their decisions, do not organize the people: they manipulate them. They do not liberate, nor are they liberated: they oppress” (Freire, 1970, p. 145). For those working in the SDOH space, reflection is a powerful tool to listen and engage, yet it can also be used to manipulate and further oppress (Boone et al., 2019). Therefore, in the vein of critical consciousness, reflection turns inward with recognition of the power and privilege of structures that have promoted oppression, marginalization, and exploitation of the vulnerable directly impacted by SDOH.

Reflection offers the opportunity to engage in power sharing and co-creating solutions. One growing effort in SDOH is to engage those with lived experience. Engaging those impacted in SDOH to ensure strategies and investments align with lived experience is a critical aspect of SDOH work. In fact, the reflections of lived experience should be as valued a data point as other forms of data on SDOH. According to the Center for Health Care Strategies Report entitled Shifting the Power Balance: Creating Health System Accountability Through Trusted Community Partnerships, there are multiple opportunities to address power to build trust including acknowledging harm, engage in transparency and seeking feedback through reflective practices (Center for Health Care Strategies, 2023). Reflection, like other aspects of professional development, is a skill. Facilitating reflection requires delving into the elements of power acknowledged by Paulo Freire and seeking to empower through reflection is important to engage in critically conscious SDOH work.

## Action

The intent of reflection in critical consciousness is to help identify and frame collective action (Diemer et al., 2016). The concepts of critical consciousness are grounded in agency in order to take a stand against oppression. Working in a SDOH ecosystem requires action to address structural concerns that impact communities. Partnerships do not evolve and grow without collective action (Solar and Irwin, 2010). Yet, SDOH are challenging and acting toward addressing them is not easy or simple. Those engaged in the work need to enter it facing these realities leaning appropriately into an asset-focused approach to stay resilient both as individuals and partners (Harrison et al., 2019). The intent of action in critical consciousness is to work toward stratification of resources toward impact (Diemer et al., 2016). In this situation, partners need to collaborate to identify the actions that need to occur in the ecosystem. These actions could be operational like identifying the social needs screener to be used by all the partners involved or agreeing to use one technology platform to ensure that data on social needs is not siloed. Decisions around consent and data governance can be made grounded in this approach as well. The actions of each ecosystem will be unique and grounded in the community needs.

Community based organizations and those with lived experience bring valuable expertise to drive meaningful action. These partners and stakeholders, if empowered, act from their expertise toward identifying what actions they can contribute to the ecosystem. In a collective action approach, the intent of this would be to ensure the partners act toward the desired outcomes of the entire ecosystem (Solar and Irwin, 2010). This means taking time to clearly identify the roles and responsibilities of each partner recognizing the opportunities and limitations of each partner in the ecosystem. It is also important to recognize that partnership work is not a light switch. Bringing partners together does not inherently produce impactful work. Intentionality and taking the time to build partner relationships has to occur (Henize et al., 2015; Puro and Kelly, 2022). This means ensuring that roles are clear and partners know their responsibility in the ecosystem. Without it, reflection will not be authentic, and the partnerships will not evolve to become high performing. There is also risk of competition and oppression that can occur when some voices are leveraged, and others are silenced. Lacking role clarity and responsibility can also lead to partners overstepping or under delivering impacting the dynamics of the ecosystem. In the case where there is lack of clarity, some of these actions may be unintentional and need to be clarified to move the ecosystem forward. The work of SDOH is too important to skip these steps leading to unintended consequences and sometimes irreparable damage.

Trust is the fundamental core for partnerships to flourish, evolve and grow. Although trust is important in all partnerships, it is especially critical in SDOH efforts due to the institutional nature of the work (Bright et al., 2017). In teamwork literature, teams have to form, storm, norm, and perform (Coleman et al., 2021). Research tells us that teams that do not storm cannot become high performing. Partnerships follow this same mantra (Kennedy-Metz et al., 2022). Building a SDOH ecosystem is grounded in collaboration and cross-sector partnerships. These partnerships all come with unique missions, individuals, and priorities. Partners must be willing to come together maximizing the assets of each organization and own that challenges will occur. Conflict is often viewed in a negative light yet is necessary to truly delve into the challenges and core issues that will arise to build the trust required to succeed (Eichbaum, 2018). Looking to the framework of critical consciousness is needed due to the complicated nature of the partnerships involved in a SDOH ecosystem.

## Motivation

Tackling SDOH are not for the faint of heart. Motivation is critical to ensure that partners feel equipped to tackle challenges as they arise. In critical consciousness, motivation is about the perception of the capacity to address injustice (Diemer et al., 2016). Partners must maintain motivation to keep moving forward in the face of challenge and conflict. Again, the best practices of interprofessional collaboration can serve as a grounding for identifying and maintaining motivation. Individuals engaged in teams are more engaged in their work (Nembhard and Edmondson, 2006). The work of Amy Edmonson discusses in detail how trust in these teams proliferates engagement (Nembhard and Edmondson, 2011). Trust was discussed as a fundamental principle in action yet is also critical to motivation.

Psychological safety is the concept that individuals can speak up without consequence (Edmonson, 1999). It is a concept that has emerged as a fundamental concept in teamwork and is critical in the SDOH ecosystem. Partners must work together to ensure that members of the partnership can speak freely, offer counter opinions, and engage in productive conflict. All of this is critical to ensure the collective action of the ecosystem is realized. Research shows that environments that are not psychologically safe are at risk to marginalize, counteracting the intended work of SDOH (Schulson et al., 2022). Speaking up, especially in a partnership model, may be considered a risk. Therefore, speaking up and bringing up different perspectives should be valued by the partners in the ecosystem not just in voice but in action through recognition, validation, and encouragement, recognizing the courage it took to voice the perspective. Giving time and space in reflection can be a good strategy to build psychological safety.

Cross-sector partners often speak and use language that other partners may not understand. It may be as simple as a technology vendor discussing data standards without taking the time to clearly define the terminology. Language and terminology can either facilitate or disrupt collaboration. In a SDOH ecosystem the diversity of partners will lead to conversations where not everyone understands the concepts unless efforts are made to educate and opportunities to ask questions are presented. Cahn (2017) discussed the Seven dirty words: hot-button language that undermines interprofessional education and practice as a foundation to demonstrate how it's not only important to communicate but recognize the power and influence words hold. In partnerships, the actions and goals must be developed in a way that bring all stakeholders to a collective understanding to ensure the same vision is held across the ecosystem. Making space to ask questions, clarify, and respond to concerns ensures that language does not act as a barrier to partnership and execution of the efforts of the ecosystem.

## Leadership

Leading and engaging in a SDOH ecosystem requires intention. Critical consciousness can provide guidance and a framework for the efforts. For those leading either from the top, the middle or on the ground should remain adaptive in their approach to the work of the SDOH ecosystem. Adaptive leadership is “composed of the set of strategies and behaviors that are used to facilitate the adaptive work, arising from individuals in the organization who foster or allow the adaptive work to occur” (Corazzini et al., 2015, p. 617). Beyond being adaptive, leaders in the work should support self-awareness of individuals in the partnership to help them collaborate more effectively. Many self-assessment tools exist to help identify preferred styles and communication. Using these tools to understand the perspectives of the members of the partnership can leverage the work and promote best practices in collaboration. Leaders can also be empowered to build partnerships and strategies based on the results leading to high performing teams and improved outcomes (Stephens et al., 2021). Self-awareness can aid in developing an ecosystem that is critical conscious in its work (Center, 2018).

Leaders in SDOH must be humble, owning and recognizing their power. They must recognize where their expertise ends and leverage community voice (Suarez-Balcazar, 2020). Understanding the nuances of power and history of an organization and its relationship with the community is of utmost importance. Two factors are incredibly important to maintaining trust as a leader in SDOH efforts−1. Transparency and 2. Follow through. Transparency in SDOH efforts including partnerships and investments is critical. In fact, transparency is fundamental as a practice of critical consciousness (Mosley et al., 2021). Follow through is equally important. When organizations make promises, the intent is to keep them and if a promise needs to be broken, transparency around that broken promise is important. It's fundamental to act against oppression as a leader. This includes recognizing the nuances and experiences around data sharing and technology along with the impact of how data gaps influence misrepresentation of certain populations and communities. Being judicious of the risks that prioritize technology or the mission of some organizations over others is essential. Creating safe spaces where community members can share, following the practices of psychological safety, is necessary for any leader in the SDOH space (Curtis et al., 2019). Leaders in this space must expect to be challenged, seek to understand, and grow as leaders in a sphere that may be unlike previous experiences. Critical consciousness offers a lens and approaches for leaders in SDOH to impact the work in ways that truly impact SDOH across systems (Pillen et al., 2020).

## Conclusion

All over the United States, efforts are being made to build SDOH and health equity strategies. The work is complex and challenging. It requires an understanding of multiple elements of health care, social care, community, data, and governance. The work is not possible alone and is supported through cross-sector partnerships and multiple funding mechanisms. Collaboration across disciplines and roles will be an important part of moving toward success. Using the best practices of critical consciousness can guide the efforts. By focusing on what each partner excels in, the partners can truly leverage the work of their ecosystem to maximize impact on SDOH.

## Data availability statement

The original contributions presented in the study are included in the article/supplementary material, further inquiries can be directed to the corresponding author.

## Author contributions

All authors listed have made a substantial, direct, and intellectual contribution to the work and approved it for publication.
